# ﻿*Scrobipalpulopsisaguilaensis* sp. nov. (Lepidoptera, Gelechiidae), the first representative of the genus discovered in the Atacama Desert, northern Chile

**DOI:** 10.3897/zookeys.1114.84509

**Published:** 2022-07-25

**Authors:** Héctor A. Vargas

**Affiliations:** 1 Universidad de Tarapacá, Facultad de Ciencias Agronómicas, Departamento de Recursos Ambientales, Casilla 6-D, Arica, Chile Universidad de Tarapacá Arica Chile

**Keywords:** DNA barcodes, florivorous larvae, Gnorimoschemini, micromoth, Neotropical, Verbenaceae

## Abstract

Genitalia morphology of a new gnorimoschemine micromoth (Lepidoptera, Gelechiidae, Gelechiinae, Gnorimoschemini) discovered in the Atacama Desert, northern Chile, fits the original description of *Scrobipalpulopsis* Povolný, 1987, a genus previously synonymized with *Scrobipalpula* Povolný, 1964. The generic assignment of the new species was assessed using a Bayesian phylogenetic analysis based on mitochondrial DNA sequences. The new species, the type species of *Scrobipalpulopsis* and another species recently transferred from this genus to *Scrobipalpula* were grouped in a monophyletic cluster distantly related to that of *Scrobipalpula*. Furthermore, an ancestral state reconstruction analysis suggested that the presence of two pairs of processes on the vinculum in the male genitalia represents a synapomorphy for the cluster of three species. Accordingly, the revalidation of *Scrobipalpulopsis***gen. rev.** (type species *Phthorimaeastirodes* Meyrick, 1931) and the reinstated combination *Scrobipalpulopsislutescella* (Clarke, 1934) **comb. rev.** are proposed. The micromoth *Scrobipalpulopsisaguilaensis***sp. nov.**, whose larvae feed on inflorescences of the Chilean endemic *Glandulariagynobasis* (Verbenaceae), is described and illustrated. Genetic divergence with congenerics was found to be 2.5–4.4% (K2P). This discovery represents the first record of *Scrobipalpulopsis* from the Atacama Desert.

## ﻿Introduction

The New World micromoth genus *Scrobipalpulopsis* Povolný, 1987 (Lepidoptera, Gelechiidae, Gelechiinae, Gnorimoschemini) was originally described to include five Neotropical and one Nearctic species, with *Phthorimaeastirodes* Meyrick, 1931, from southern Argentina and southern Chile, as the type species (Povolný 1987). An elongated gnathos with a serrated apical ledge, serration on the ventral wall of the phallus and vinculum with two pairs of processes in the male genitalia, and a long colliculum on the membranous ductus bursae in the female genitalia were highlighted as morphological characters to differentiate representatives of this genus from those of the remarkably similar *Scrobipalpula* Povolný, 1964 (Povolný 1987). Two Neotropical (Povolný 1994) and one Nearctic (Powell and Povolný 2001) species were subsequently added to *Scrobipalpulopsis*. The genitalia morphology of the former two (Clarke 1965; Povolný 1990) agrees with the original genus description (Povolný 1987), whereas the latter species, originally described as *Scrobipalpulopsislycii* Powell & Povolný, 2001, was referred as “an isolated taxon with distant relations to other members of the genus” due to a very deep and broad medial excision on the vinculum (Powell and Povolný 2001). As part of the checklist of the fauna of Gelechiidae of America north of Mexico, *Scrobipalpulopsis* was synonymized with *Scrobipalpula* and the two Nearctic representatives of the former [*lycii* and *lutescella* (Clarke, 1934)] were transferred to the latter (Lee et al. 2009).

Although the micromoth fauna of the Atacama and Puna biogeographic provinces of northern Chile has been little explored, recent discoveries suggest that plants native to the extremely arid environments of this part of South America host overlooked species of several micromoth families, including Gelechiidae (Vargas 2019, 2020). As part of surveys for Lepidoptera in this area, a few micromoths were recently reared from larvae collected on inflorescences of a native plant in the Atacama Desert. Subsequent examination of their genitalia revealed that these specimens represent a new gelechiid species whose morphology agrees with the original description of *Scrobipalpulopsis* (Povolný 1987). The aim of this study is to assess the generic assignment of this new species using a phylogenetic analysis based on mitochondrial DNA sequences and to provide a formal taxonomic description for it.

## ﻿Materials and methods

### ﻿Specimens

The adult specimens examined in this study were obtained in October, 2021 from larvae collected in September, 2021 on inflorescences of *Glandulariagynobasis* (Wedd.) N. O’Leary & P. Peralta (Verbenaceae) in Cuesta El Águila (18°29'08"S, 69°51'55"W), at about 1950 m elevation in the Cardones ravine, Arica Province, northern Chile. Two larvae were kept in 95% ethanol at –20 °C until DNA extraction. The abdomen of each adult was removed and placed in hot KOH 10% for a few minutes for genitalia dissection. Genitalia were stained with Eosin Y and Chlorazol Black and mounted on slides with Euparal. The photo of the adult was taken with a Leica Flexacam C1 digital camera attached to a Leica M125 stereomicroscope. Photos of the genitalia were taken with a Leica MC170 HD digital camera attached to a Leica DM1000 LED light microscope. Each image was constructed with about 5–20 photos assembled with the software Helicon Focus 8. Terminology of Huemer and Karsholt (2010) is followed for the genitalia. The specimens studied are deposited in the “Colección Entomológica de la Universidad de Tarapacá” (IDEA), Arica, Chile.

### ﻿DNA extraction, sequencing, and analysis

Genomic DNA was extracted from two larvae using the QIAamp Fast DNA Tissue Kit, following the manufacturer’s instructions, and sent to Macrogen Inc. (Seoul, South Korea) for purification, PCR amplification and sequencing of the barcode region (Hebert et al. 2003) using primers LCO1490 and HCO2198 (Folmer et al. 1994) with a PCR program of 5 min at 94 °C, 35 cycles of 30 s at 94 °C, 30 s at 47 °C, 1 min at 72 °C and a final elongation step of 10 min at 72 °C. Phylogenetic analyses based on this molecular marker have been used successfully for generic assignment of species of different families of Lepidoptera (e.g., Moreira et al. 2012; San Blas et al. 2021), including Gelechiidae (Metz et al. 2019; Corley et al. 2020). One full-length (658 bp) DNA barcode sequence of each species of *Scrobipalpula*, one of the type species of each Gnorimoschemini genus represented in the Neotropical region, one of the type species of *Gnorimoschema* Busck, 1900 and one of the type species of *Gelechia* Hübner, [1825] (Gelechiini) were downloaded from BOLD (Ratnasingham and Hebert 2007) to be included in further analyses (Table 1). The software MEGA11 (Tamura et al. 2021) was used to perform alignment and to estimate divergence of the sequences with the ClustalW and Kimura 2-Parameter (K2P) methods, respectively. The Xia test of substitution saturation (Xia et al. 2003) was performed with the software DAMBE7 (Xia 2018) to evaluate the utility of the alignment for phylogenetic inference. A consensus tree was reconstructed using Bayesian inference in the software MrBayes 3.2 (Ronquist et al. 2012) with GTR+I+Γ as substitution model and four Markov Chain Monte Carlo (MCMC) chains run for 1,000,000 generations, sampling every 500 generations, with the first 25% sampled trees discarded as burn-in. Branch support was estimated using posterior probability (PP) values. The unrooted tree was visualized in FigTree (Rambaut 2014) to root on the representative of *Gelechia*, following the phylogeny of Gelechiidae proposed by Karsholt et al. (2013).

**Table 1.** DNA barcode sequences (658 bp length) used in the molecular analysis.

Species	BOLD accession	GenBank accession	Country
*Eurysaccamelanocampta* (Meyrick, 1917)	LNAUU4648-15		Bolivia
*Gelechiarhombella* (Denis & Schiffermüller, 1775)	ABOLA335-14	MN805882	Italy
*Gnorimoschemagallaesolidaginis* (Riley, 1869)	RDLQD617-06		Canada
*Keiferialycopersicella* (Walsingham, 1897)	BBLOE243-11		USA
*Scrobipalpopsispetasitis* (Pfaffenzeller, 1867)	FBLMT476-09	HM422119	Germany
*Scrobipalpulaantiochia* Powell & Povolný, 2001	LGSMD329-05		USA
*Scrobipalpulamanierreorum* Priest, 2014	ALLEP078-13		Canada
*Scrobipalpulapsilella* (Herrich-Schäffer, 1854)	ABOLA839-15	MN804394	Austria
*Scrobipalpulasacculicola* (Braun, 1925)	LEPNF719-14		Canada
*Scrobipalpulatussilaginis* (Stainton, 1867)	CGUKD141-09		United Kingdom
*Scrobipalpulawilsoni* Vargas, 2019	GBMNB43086-20	MK749395	Chile
*Scrobipalpulopsisaguilaensis* sp. nov.		ON007244	Chile
*Scrobipalpulopsisaguilaensis* sp. nov.		ON007245	Chile
*Scrobipalpulopsislutescella* (Clarke, 1934)	CHIP167-12	KT140769	Canada
*Scrobipalpulopsisstirodes* (Meyrick, 1931)	NCNGS022-17		Argentina
*Symmetrischematangolias* (van Gyen, 1913)	GBMIN80431-17	KX443106	Peru
*Phthorimaeaoperculella* (Zeller, 1873)	ANICX1239-11	KF387868	Australia

### ﻿Ancestral state reconstruction

The presence of two pairs of processes on the vinculum in the male genitalia was highlighted by Povolný (1987) as the main morphological feature to differentiate members of *Scrobipalpulopsis* from those of *Scrobipalpula*, as in the latter genus the vinculum has only one pair of processes. To assess a potential synapomorphy for *Scrobipalpulopsis*, an analysis of ancestral state reconstruction for the character “number of pairs of processes on the vinculum” with two states, one pair (0) and two pairs (1), was performed using parsimony in the software Mesquite 3.70 (Maddison and Maddison 2021). The Bayesian consensus tree here obtained was used for character mapping.

## ﻿Results

### ﻿Molecular analysis and ancestral state reconstruction (Figs 1, 2)

Two DNA barcode sequences (ON007244, ON007245) with 0.5% (K2P) divergence between them were obtained. The alignment of 657 bp length included 17 sequences with no evidence of stop codons. Substitution saturation was not detected (ISS < ISS.C; p < 0.001), indicating that the alignment was suitable for phylogenetic analysis. The nearest sequence to those of the new species was that of the type species of *Scrobipalpulopsis* (i.e. *stirodes*) with 2.5–2.6% (K2P) divergence. The Bayesian analysis found a monophyletic cluster of *Scrobipalpula* that excluded *stirodes*, the Nearctic *lutescella* and the new species. These three species were grouped in a monophyletic cluster into which the new species was sister to *stirodes*. Furthermore, the result of the ancestral state reconstruction analysis suggested that the presence of two pairs of processes on the vinculum in the male genitalia represents a synapomorphy for this group of three species. Accordingly, the revalidation of the genus *Scrobipalpulopsis* Povolný, 1987 and the reinstatement of the combination *Scrobipalpulopsislutescella* (Clarke, 1934) are proposed and the new species is assigned to this genus.

**Figure 1.**
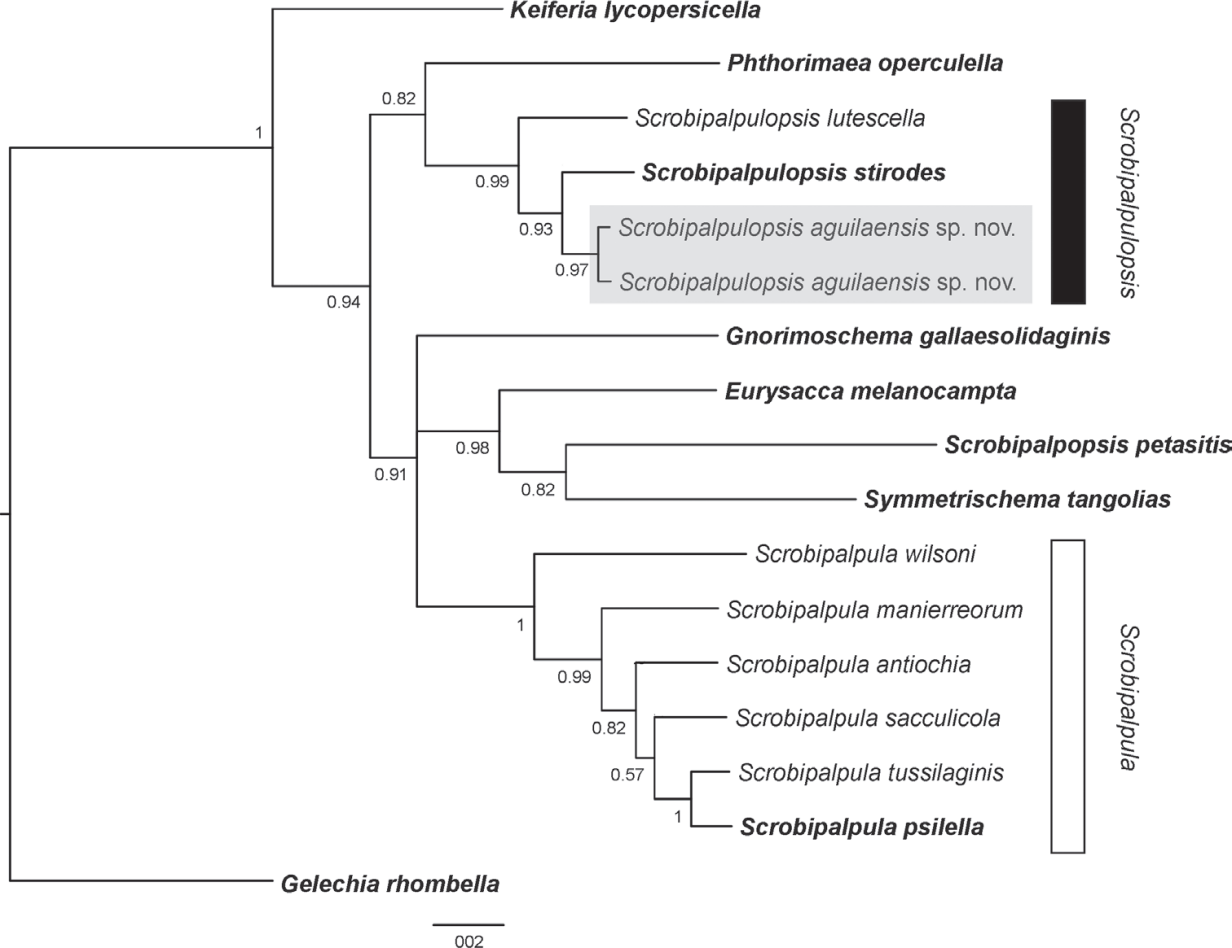
Bayesian consensus tree of *Scrobipalpulopsisaguilaensis* sp. nov. (grey rectangle) and representatives of Gnorimoschemini based on mitochondrial DNA sequences. The type species of *Gelechia* Hübner, [1825] (Gelechiini) was used to root the tree. Type species in bold. Posterior probability values indicate node support.

**Figure 2.**
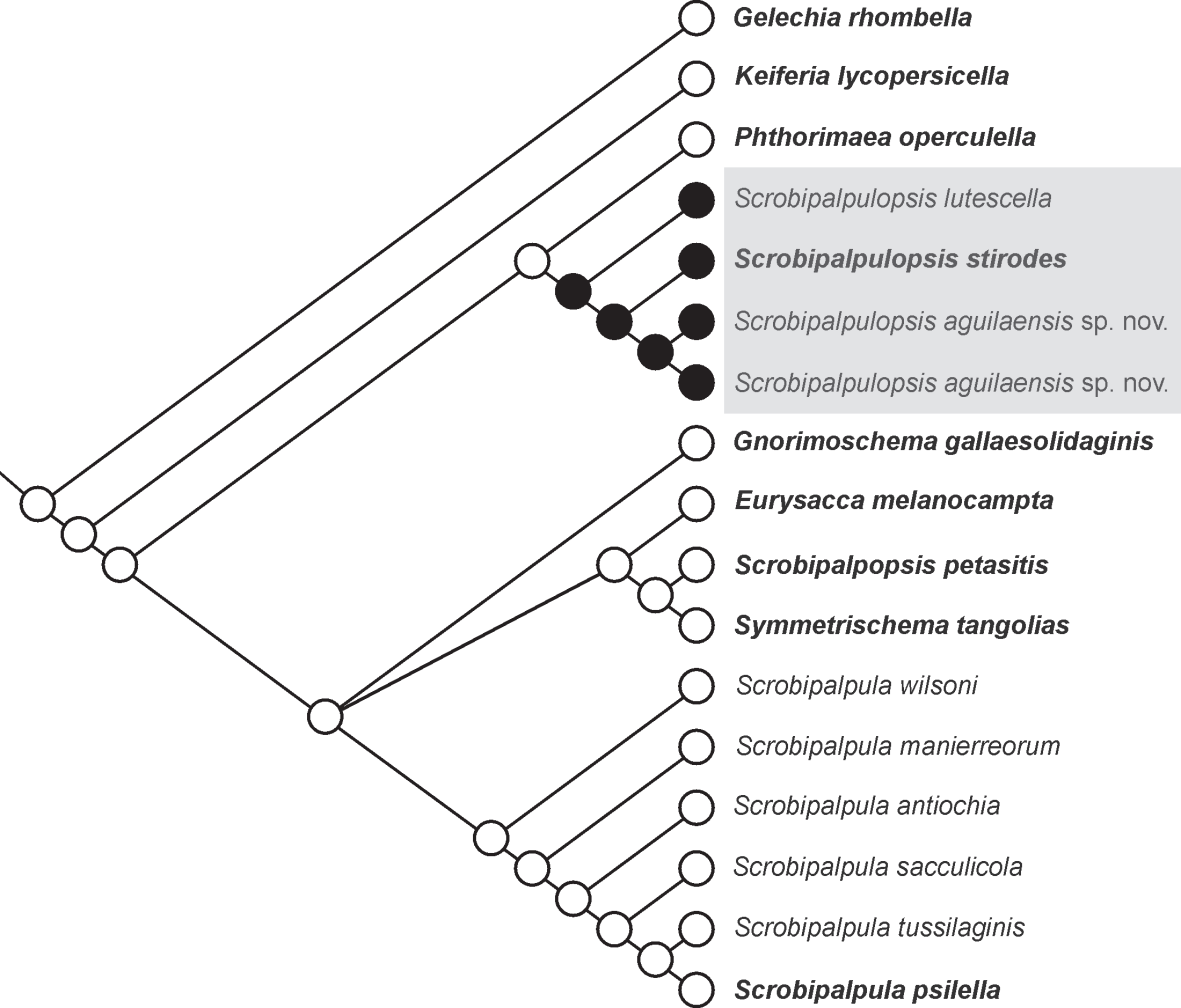
Ancestral state reconstruction using parsimony for the character “number of pairs of processes on the vinculum” with two states, one pair (open circle) and two pairs (closed circle). Type species in bold.

### ﻿Taxonomy

The following checklist is based on Povolný (1994) and Powell and Povolný (2001).

#### ﻿ Scrobipalpulopsis

Taxon classificationAnimaliaLepidopteraGelechiidae

Povolný, 1987 gen. rev.

6CF092A2-03EF-5774-AC02-3DE9A520841B

https://zoobank.org/FACA85EB-1979-4630-87A3-850591FF8FF8

##### Type species.

*Phthorimaeastirodes* Meyrick, 1931, by original designation

*Scrobipalpulopsisaguilaensis* sp. nov.

*Scrobipalpulopsisdispar* Povolný, 1990

*Scrobipalpulopsisfallacoides* Povolný, 1987

*Scrobipalpulopsisfallax* Povolný, 1987

*Scrobipalpulopsishemilitha* (Clarke, 1965)

*Scrobipalpulopsislutescella* (Clarke, 1934) comb. rev.

*Scrobipalpulopsispraeses* Povolný, 1987

*Scrobipalpulopsissimulatrix* Povolný, 1987

*Scrobipalpulopsisstirodes* Meyrick, 1931

#### ﻿ Scrobipalpulopsis aguilaensis sp. nov.

Taxon classificationAnimaliaLepidopteraGelechiidae

0A823647-03F8-5BF4-B231-8EB44ED487DC

https://zoobank.org/B914597C-3CF5-4FB6-9EF3-5418C3BAEF6E

**Figure 3.**
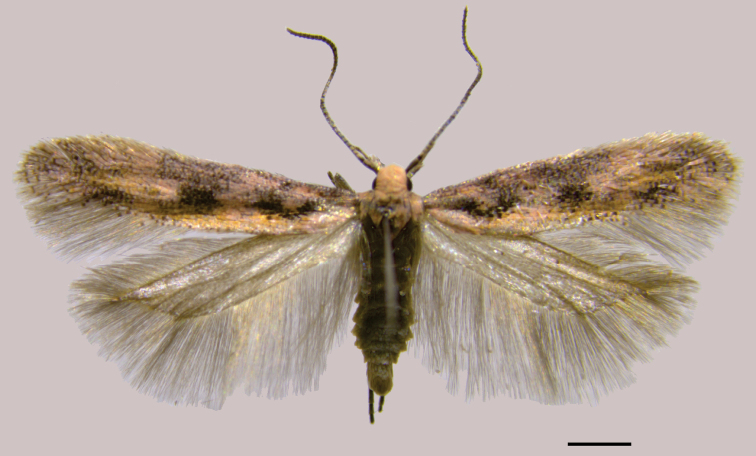
Holotype male of *Scrobipalpulopsisaguilaensis* sp. nov., dorsal. Scale bar: 1 mm.

**Figure 4.**
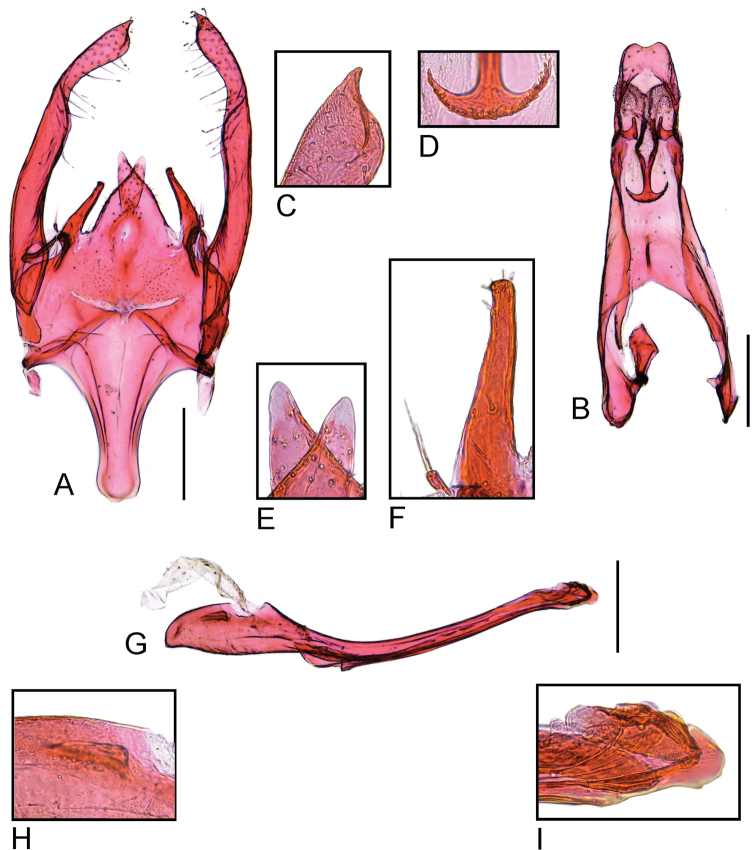
Male genitalia of *Scrobipalpulopsisaguilaensis* sp. nov. **A** valvae, vinculum and saccus, ventral **B** tegumen, uncus and gnathos, ventral **C** apex of left valva, ventral **D** distal part of gnathos showing finely serrated margin **E** tip of the left (right) and right (left) medial processes of the vinculum, ventral **F** left lateral process of the vinculum (right) and left sacculus (left), ventral **G** phallus, lateral **H** left lateral process of the coecum, lateral **I** tip of the phallus, lateral. Scale bar: 0.2 mm.

**Figure 5.**
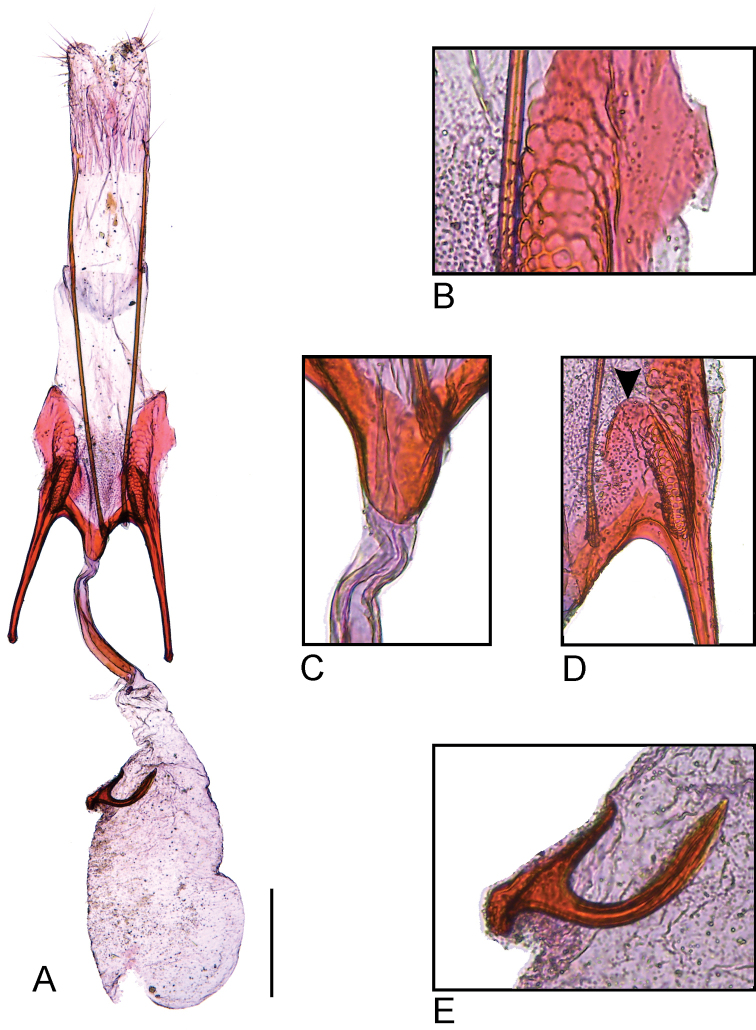
Female genitalia of *Scrobipalpulopsisaguilaensis* sp. nov. **A** female genitalia, ventral **B** detail of right longitudinal sclerite with honeycomb sculpture and membranous area with microtrichia of segment VIII, ventral **C** antrum, ventral **D** detail of the flat, semicircular posterior projection of the apophysis anterioris, ventral; black arrow indicates posterior margin **E** detail of signum, ventral. Scale bar 0.2 mm.

**Figure 6.**
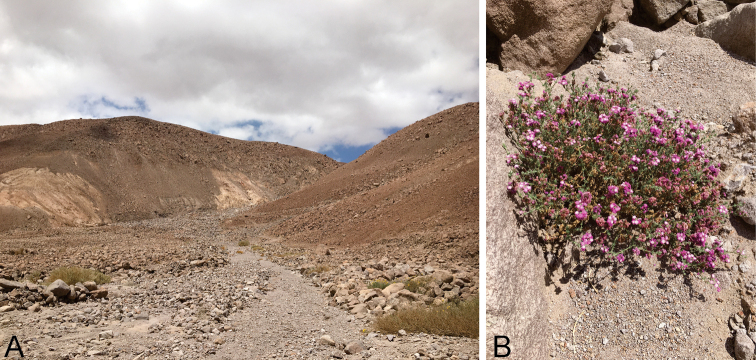
Habitat and host plant of *Scrobipalpulopsisaguilaensis* sp. nov. **A** habitat at the type locality, Cuesta el Águila, Cardones ravine, Arica Province, northern Chile **B** host plant, *Glandulariagynobasis* (Verbenaceae), at the type locality.

Figs 1 , 2 , 3 , 4 , 5 , 6

##### Type locality.

Chile, Arica, Cardones, Cuesta El Águila (18°29'08"S, 69°51'55"W), 1950 m elevation.

##### Type material.

***Holotype***: Chile • ♂; Arica, Cardones / Cuesta El Águila [18°29'08"S, 69°51'55"W] / October 2021 / H.A. Vargas leg. / ex-larva inflorescence / *Glandulariagynobasis* / September 2021 / “Holotype / *Scrobipalpulopsis* / *aguilaensis* / Vargas” [red handwritten label] / IDEA-LEPI-2022-001 / HAV-1526 [genitalia slide]. Specimen and genitalia slide deposited at IDEA. ***Paratypes***: same data as for the holotype • 3 ♂♂, 2 ♀♀; IDEA-LEPI-2022-002 to IDEA-LEPI-2022-006; HAV-1516, 1517, 1523, 1524, 1525 [genitalia slides]. Specimens and genitalia slides deposited at IDEA.

##### Diagnosis.

*Scrobipalpulopsisaguilaensis* sp. nov. is recognized by the pale pink ground color of the forewing and the pointed apex of the valva in the male genitalia. The genitalia of *S.aguilaensis* sp. nov. resemble those of *S.stirodes*, which is also its nearest congener based on DNA barcodes. However, the two species can be easily distinguished, as the latter has greyish ground color on the forewing and the apex of the valva slightly dilated. Furthermore, *S.stirodes* has a well-developed flat edge on the valva, the lateral process of the vinculum is longer than the medial one, the saccus is almost as long as wide and the phallus has a longitudinal serrated ledge in the male genitalia. In contrast, the valva lacks a flat edge, the medial process of the vinculum is longer than the lateral one, the saccus is longer than wide and the longitudinal ledge of the phallus lacks serrations in the male genitalia of *S.aguilaensis* sp. nov. In the female genitalia, the antrum of *S.stirodes* is membranous, while the antrum of *S.aguilaensis* sp. nov. is sclerotized.

##### Description.

**Male** (Fig. 3). Forewing length 4.7–5.1 mm. ***Head*.** Vertex pale pink; frons creamy white; labial palp pale pink mixed with creamy white and greyish brown; haustellum creamy white basally; antenna with scape and pedicel greyish brown, flagellum with transverse stripes greyish brown and creamy white. ***Thorax*.** Pale pink dorsally, creamy white latero-ventrally; legs with coxa and femur creamy white with scattered pale pink, tibia and tarsus mostly greyish brown with scattered creamy white and pale pink; forewing ground color pale pink, with mostly scattered greyish brown forming irregular dark spots on proximal two-thirds, yellowish brown near anal margin, fringe mostly greyish brown; hindwing and fringe greyish brown. ***Abdomen*.** Mostly greyish brown with scattered creamy white and pale pink.

***Male genitalia*** (Fig. 4). Tegumen narrow, about three times as long as wide in the middle, anterior margin broadly concave. Uncus flat, rectangular, posterior margin excavated in the middle. Gnathos Y-shaped, tip of mesial arm transversely expanded with finely serrated margin; surface of culticula covered with microtrichia. Saccus linguiform, slightly longer than half the length of valva, posterior margin slightly convex, anterior projection narrow with slightly concave lateral margin and rounded tip. Valva narrow, finger-like, slightly shorter than tegumen and uncus, apex pointed. Sacculus small, cylindrical, with two setae at apex. Vinculum with two pairs of processes; medial one flat with rounded tip; lateral one cylindrical, well-sclerotized with a few small apical setae, shorter than medial process. Phallus cylindrical, narrow, curved, slightly longer than tegumen and uncus; ventral ledge with protrusion near coecum, slightly variable in shape among specimens; coecum slightly swollen with pair of small lateral processes; apex with small flat sclerite.

**Female.** Similar to male in size and wing maculation.

***Female genitalia*** (Fig. 5). Papilla analis lobe-like with a few hair-like setae. Apophysis posterioris narrow, straight, about four times the length of papilla analis. Apophysis anterioris straight, about 1/3 the length of apophysis posterioris, with flat, semicircular posterior projection medially continuous with the antrum. Segment VIII ventrally with two irregularly sculptured longitudinal sclerites anteriorly continuous with the respective apophysis anterioris, medially separated by sinus vaginalis with microtrichia. Antrum conical, well-sclerotized. Ductus bursae with short membranous part posteriorly, colliculum elongate, about three-fourths length of ductus bursae. Corpus bursae membranous, elongate, pear-shaped, about 2.5 times the length of ductus bursae; a single, well-sclerotized strongly curved signum; inception of ductus seminalis near posterior end of corpus bursae.

##### Etymology.

The specific epithet is derived from the type locality.

##### Distribution

**(Fig. 6A).***Scrobipalpulopsisaguilaensis* sp. nov. is currently known only from the type locality, Cuesta El Águila (18°29'08"S, 69°51'55"W), at about 1950 m elevation in the Cardones ravine, Atacama Desert of northern Chile.

##### Host plant

**(Fig. 6B).** Larvae of *S.aguilaensis* sp. nov. feed on inflorescences of *Glandulariagynobasis* (Wedd.) N. O’Leary & P. Peralta (Verbenaceae), a plant endemic to the northernmost part of Chile growing between 1900–4000 m elevation (Rodriguez et al. 2018).

## ﻿Discussion

Generic assignment of the species of Neotropical Gnorimoschemini can be particularly difficult due to instability of genus-level circumscriptions (Corro Chang and Metz 2021). Phylogenetic analyses of mitochondrial DNA sequences have provided accurate generic assignment for species of several families of Lepidoptera (Lees et al. 2010; Moreira et al. 2012; Davis et al. 2020; San Blas et al. 2021; Nupponen and Sihvonen 2022). In the case of Gelechiidae, the agreement between phylogenetic analysis of mitochondrial DNA sequences and morphology has recently supported the recognition of new genera (Metz et al. 2019; Corley et al. 2020). The result of the Bayesian analysis of the present study provides support for the revalidation of *Scrobipalpulopsis*, because its type species was found to be not monophyletic with that of *Scrobipalpula*. This result agrees with morphology, as species of *Scrobipalpula* are recognized by a shovel-shaped gnathos and vinculum with one pair of processes in the male, and a ring-shaped colliculum in the female (Huemer and Karsholt 1998, 2010), while *S.stirodes* has an elongate gnathos with a finely serrated apex and a vinculum with two pairs of processes in the male and an elongate colliculum in the female (Povolný 1987). The result also supports the congeneric status of *S.stirodes* with *S.aguilae* sp. nov. and the Nearctic *S.lutescella*, because the three species were grouped with high support in a monophyletic cluster. Furthermore, the ancestral state reconstruction identified the presence of two pairs of processes on the vinculum in the male genitalia, a state shared by these three species, as a synapomorphy for *Scrobipalpulopsis*. However, further studies are needed for a better morphological delineation of this genus.

Although the Bayesian analysis found high support for the tribe Gnorimoschemini, relationships between the Gnorimoschemini genera were poorly resolved, as indicated by low supports. For instance, *Phthorimaeaoperculella* (Zeller, 1873) was sister to *Scrobipalpulopsis*, but only with 0.82 (PP). The results of maximum parsimony and maximum likelihood analyses (see Suppl. material 1) were similar to that of the Bayesian consensus tree, with high support for the distantly related clusters of *Scrobipalpulopsis* and *Scrobipalpula* and poorly resolved relationships between genera, but with a few differences in the number of polytomies. Better taxon sampling and analysis of additional molecular markers would be valuable to resolve the relationships between the genera of Neotropical Gnorimoschemini. Morphological characters must be also assessed using cladistics analysis to determine synapomorphies for each genus following the recent example provided by Corro Chang and Metz (2021).

The discovery of larvae of *S.aguilaensis* sp. nov. feeding on inflorescences of *G.gynobasis* adds Verbenaceae as a new host plant family for *Scrobipalpulopsis*. Previous host plant records for *Scrobipalpulopsis* were restricted to *S.lutescella*, whose larvae feed on flowers of *Castilleja* spp. (Orobanchaceae) (Clarke 1934; Powell and Povolný 2001; Nazari and Landry 2012). The association of *Scrobipalpulopsis* with a representative of Solanaceae was recorded by Powell and Povolný (2001). However, this record was based on *lycii*, a species currently included in *Scrobipalpula* (Lee et al. 2009). Host plant records can be useful to guide surveys to characterize distribution ranges of little-known species of phytophagous insects. The type locality of *S.aguilaensis* sp. nov. currently represents the only distribution record for this micromoth. However, this site (at about 1950 m) is at the lower limit of the wide elevational range of the host plant, which extends to 4000 m in the Andes (Rodriguez et al. 2018). Surveys for larvae of *S.aguilaensis* sp. nov. on inflorescences of *G.gynobasis* along this altitudinal gradient would be valuable to verify whether this micromoth occurs at higher elevations.

Despite the extreme aridity of the Atacama Desert, which is one of the oldest regions in the world under continuously arid conditions (Clarke 2006), its native plants support populations of native species of Lepidoptera that have remained overlooked until recently (Davis et al. 2020; Espinoza-Donoso et al. 2020; Vargas 2021). It is hoped that the discovery of *S.aguilaensis* sp. nov. will encourage further surveys to understand better and protect the still insufficiently studied biodiversity harbored by the extremely arid and fragile environments of the Atacama Desert.

## Supplementary Material

XML Treatment for Scrobipalpulopsis

XML Treatment for Scrobipalpulopsis aguilaensis

## Supplementary materials

Supplementary material 1 Figures S1, S2 Data type Phylogenetic trees Explanation note Maximum parsimony and maximum likelihood trees of *Scrobipalpulopsisaguilaensis* sp. nov. and representatives of Gnorimoscheminibased on mitochondrial DNA sequences. https://binary.pensoft.net/file/719138This dataset is made available under the Open Database License (http://opendatacommons.org/licenses/odbl/1.0/). The Open Database License (ODbL) is a license agreement intended to allow users to freely share, modify, and use this Dataset while maintaining this same freedom for others, provided that the original source and author(s) are credited.Héctor A. Vargas
