# Supplementary material for: ﻿Scrobipalpulopsisaguilaensis sp. nov. (Lepidoptera, Gelechiidae), the first representative of the genus discovered in the Atacama Desert, northern Chile
Source: Zookeys. 2022 Jul 25;1114:105–19. doi: 10.3897/zookeys.1114.84509 (PMC9848691; doi:10.3897/zookeys.1114.84509)
Supplement: Supplementary material 1 — Figures S1, S2 [file zookeys-1114-105_article-84509__-s001.pdf]

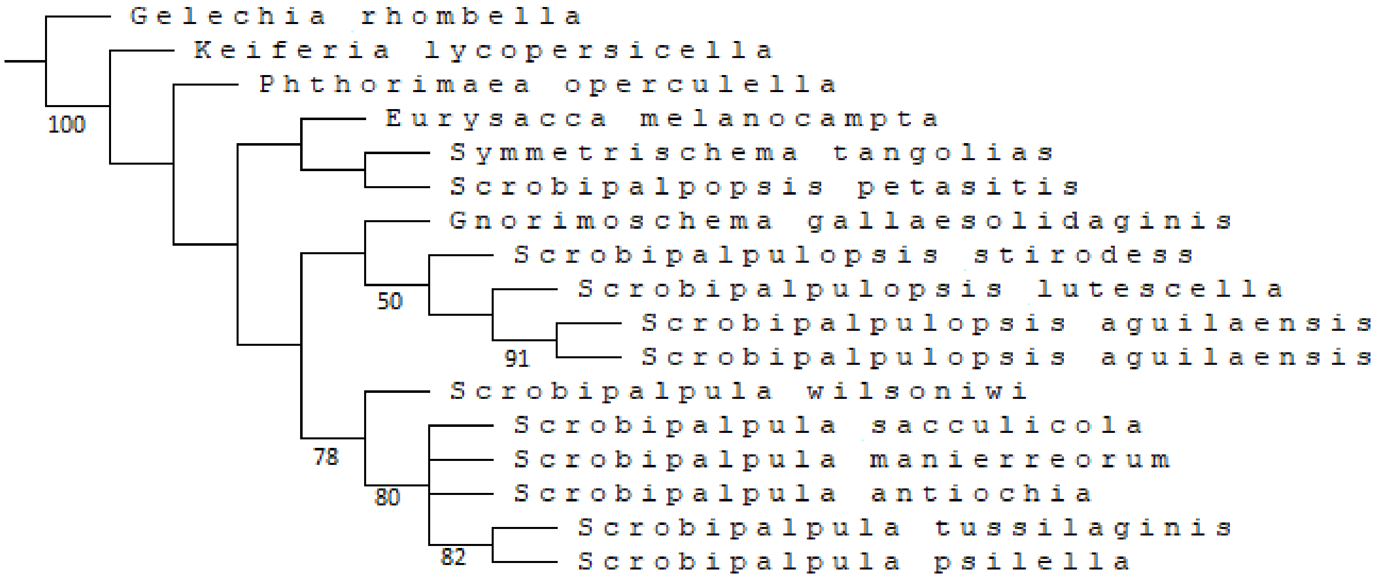

Strict consensus of two most parsimonious trees (453 steps) of *Scrobipalpulopsis aguilaensis* sp. nov. and representatives of Gnorimoschemini based on mitochondrial DNA sequences. Bootstrap values ≥ 50 are shown.

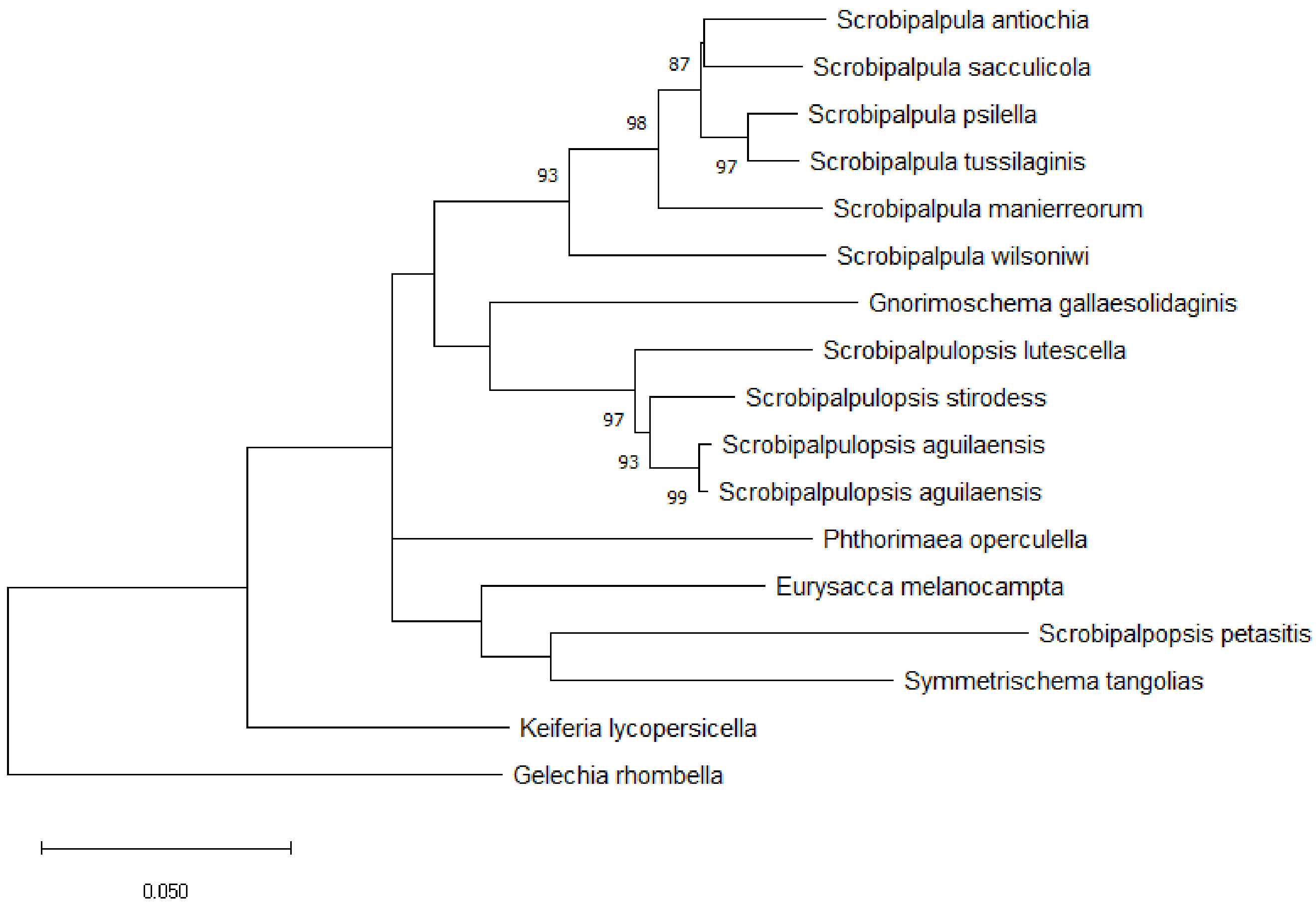

Maximum likelihood tree of *Scrobipalpulopsis aguilaensis* sp. nov. and representatives of Gnorimoschemini based on mitochondrial DNA sequences. Bootstrap values >85 are shown.
